# A discussion on a suspected case with EGPA after maintenance hemodialysis for 5 years and related literature analysis: A case report

**DOI:** 10.1097/MD.0000000000039856

**Published:** 2024-09-20

**Authors:** Lingshan Zhao, Chenli Zhang

**Affiliations:** a Department of Nephrology, The Second People’s Hospital of Yibin, Yibin, Sichuan, China.

**Keywords:** ANCA, asthma, EGPA, maintenance hemodialysis, rash

## Abstract

**Rationale:**

Pathological featuring by necrotizing granulomatous inflammation of peripheral blood and tissues with increased eosinophils infiltrating small and medium vessels, eosinophilic granulomatosis with polyangiitis (EGPA), a family of rare antineutrophil cytoplasmic antibody (ANCA) associated with systemic vasculitis. With low morbidity, diverse clinical manifestations, and difficult early diagnosis, the majority of patients are confirmed after multiple organ damages, thus missing the best treatment time and having a poor prognosis. About 25% to 30% of EGPA cases have been reported to suffer from the renal disease, and there are few studies on EGPA complicated with kidney damage, most of them on ANCA-positive patients. Generally, the initial diagnosis of EGPA on maintenance hemodialysis is even rare. We report a case of a patient with maintenance hemodialysis for 5 years and then was diagnosed with EGPA.

**Patient concerns:**

The female patient, 54-year-old, having maintenance hemodialysis for 5 years consecutively, was hospitalized for the recurring rash in the past 3 years and then exacerbation in the last 2 months. With the previous history of bronchial asthma having attacked frequently recently, it could be observed from peripheral blood that the eosinophils increased, from the cardiac color ultrasound that it was prone to eosinophilic endocarditis, from 5 tests for vasculitis that P-ANCA and MPO-AB were positive.

**Diagnoses:**

The patient’s onset is renal dysfunction, with maintenance hemodialysis for 5 years, recurrent lung infections, combined with eye lesions, scattered skin rashes, P-ANCA positive, MPO-AB positive, asthma present, eosinophil absolute value 1.60 × 10^9^/L, total score >6 points, diagnosis considering EGPA.

**Interventions:**

Due to multiple organ damage, the patient received treatment with a combination of steroids and cyclophosphamide.

**Outcomes:**

After 2 days, the patient’s rash significantly darkened compared to before, wheezing improved, and eosinophils returned to normal levels.

**Lessons:**

The ANCA test shall be put on the high agenda for patients presenting with kidney failure at first. Meanwhile, the neglected immune monitoring for patients with dialysis tells us that it is of great significance for this kind of patient to have immune monitoring in the early diagnosis of EGPA.

## 1. Introduction

Pathological featuring by necrotizing granulomatous inflammation of peripheral blood and tissues with increased eosinophils infiltrating small and medium vessels, eosinophilic granulomatosis with polyangiitis (EGPA), a family of rare antineutrophil cytoplasmic antibody (ANCA) associated with systemic vasculitis^,[1]^ causes diseases including asthma, eosinophilia, heart failure, renal impairment, and peripheral neuropathy^.[2]^ With low morbidity of 1 to 4.2 per million annually^.[3]^, diverse clinical manifestations, and difficult early diagnosis, the majority of patients are confirmed after multiple organ damages, thus missing the best treatment time and having a poor prognosis. 25% to 30% of EGPA cases have been reported to suffer from the renal disease,^[4]^ and there are few studies on EGPA complicated with kidney damage, most of them on ANCA-positive patients. Generally, the initial diagnosis of EGPA on maintenance hemodialysis is even rare. A patient with maintenance hemodialysis for 5 years was admitted to our department and then was diagnosed with EGPA. The paper aims to be helpful for clinicians in the clinical manifestations, auxiliary examination and treatment of EGPA through the review, and analysis of the diagnosis and treatment of the case.

## 2. Clinical data

### 2.1. General information

A 54-year-old female patient was admitted to our hospital for “maintenance hemodialysis for 5 years, recurrent limb rash for 3 years, and aggravation for 2+ months.” As unexplained renal failure was diagnosed because of bilateral lower extremity edema, heart failure, and shortness of breath, the patient received regular hemodialysis treatment lasting for 5 years. There was no rash, asthma, and other symptoms, and increased eosinophil during the first 2 years of hemodialysis, while rash on the limbs existed 2 years later, especially evident in summer, and subsided completely in spring and winter without systematic treatment. Salmeterol was taken continuously after diagnosis of bronchial 3 years ago. Asthma has frequently been attacked people recently without special personal or family disease history.

**Figure 1.**
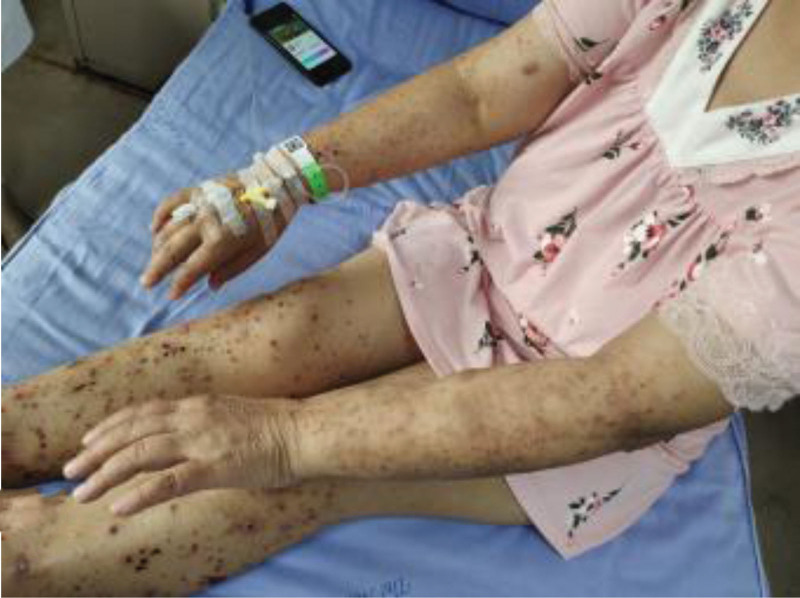
Papules and blood scabs on the patient’s limbs.

Physical examination: hyperemia in both eyes, no obvious dry or moist rales in both lungs, normal heart rhythm, no obvious murmurs in each valve, slightly distended abdomen, no tenderness or muscle tension in the whole abdomen, multiple papules, blood scabs as well as scratches can be seen in the exposed parts of the limbs (Fig. 1A).

### 2.2. Auxiliary inspection

It was shown in the blood routine examination that white blood cells were 9.76 × 10^9^/L, the absolute value of eosinophil 1.60 × 10^9^/L, the eosinophil rate 16.40%, hemoglobin 121.00 g/L, platelet 237.00 × 10^9^/L; C-reactive protein 13.84 mg/L, hypersensitive C-reactive protein > 10.00 mg/L, procalcitonin 1.00 ng/mL, erythrocyte sedimentation rate 74.00 mm/h, IGE 752 IU/mL, IgG 18.69 g/l, natriuretic peptide 5504 pg/mL, creatine kinase isoenzyme MB2.15 ng/mL, myoglobin 274.3 ng/mL, hypersensitive troponin T quantification 28.25 pg/mL, and liver function was normal. The renal function suggested creatinine was 1083 μmol/L, calcium 2.64 mmol/L, magnesium 1.11 mmol/L, phosphorus 1.47 mmol/L. No obvious abnormalities were observed in hepatitis B, hepatitis C, syphilis, and AIDS. Autoimmune antibody spectrum showed weakly positive proliferation cell nuclear antigen and weakly positive antibody (AMA). ANA: 1:100 positive; 1:320 positive. ECG displayed sinus rhythm and the changes of ST-T.

The 5 test items for vasculitis suggested positive P-ANCA and MPO-Ab.

Bone marrow smears revealed an increase in the proportion of eosinophils.

No abnormality of parasite-related antibody and Epstein–Barr virus was observed.

Vascular ultrasound showed no definite abnormality.

**Figure 2.**
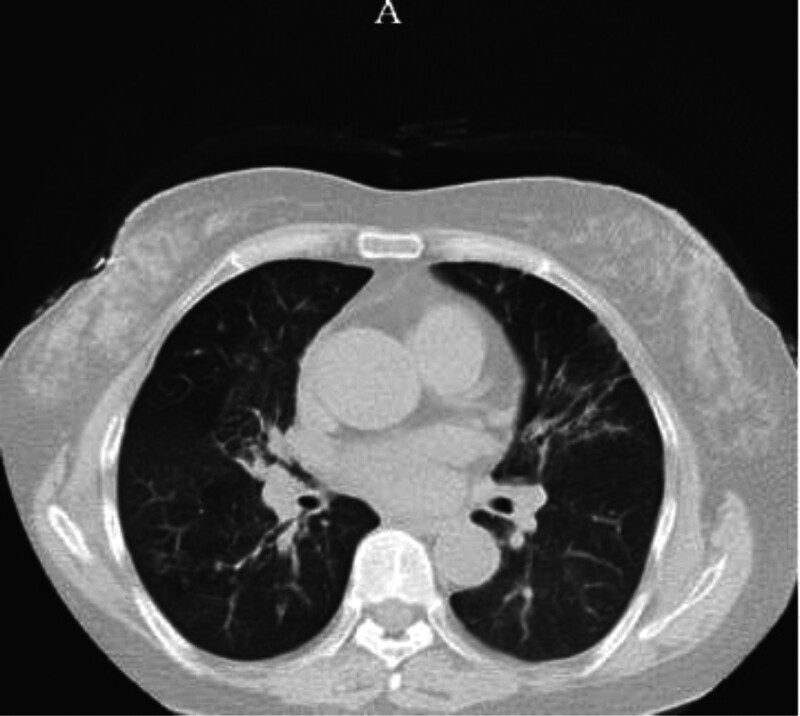
Multi-sheet and cord-like increased density shadows in both lungs, heterogeneous emphysema, and slightly dilated bronchi.

It was shown in chest CT that multilobed segmental and cord-shaped dense shadows in both lungs, mainly in the lower lobes of both lungs, taking pneumonia with most lesions being chronic, uneven emphysema in both lungs and slightly dilated multilobed segmental bronchus in both lungs into consideration (Fig. 2A).

**Figure 3.**
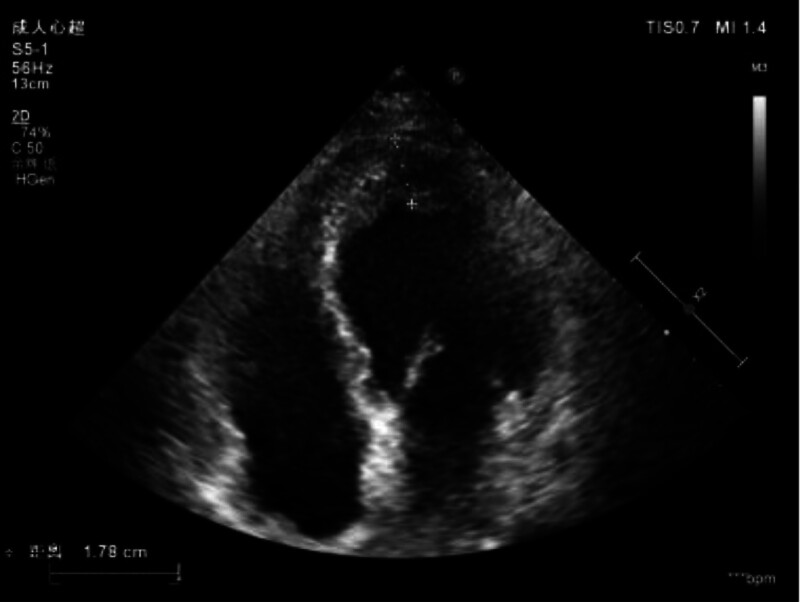
Thickening of the apical area of the left ventricle with reduced echogenicity.

Heart color Doppler ultrasound: Anterior–posterior diameter of left atrium: 35 mm, left ventricular short-axis end-diastolic diameter: 52 mm, posterior wall thickness: 9 mm. The left ventricular apex is thickened and the echo is reduced, about 18 mm thick, mild regurgitation was detected below the valve orifice when the aortic valve was closed. Tricuspid valve and mild regurgitation, the regurgitation velocity is 2.89 m/s, the pressure difference is 33 mmHg, and the estimated pulmonary artery systolic pressure is 36 mmHg. Diagnosis: Enlargement of the left atrium and ventricle. Thickening of the apex of the left ventricle and decreased echo: eosinophilic endocarditis? Aortic valve degeneration with regurgitation (mild). Tricuspid regurgitation (mild) (Fig. 3A).

**Figure 4.**
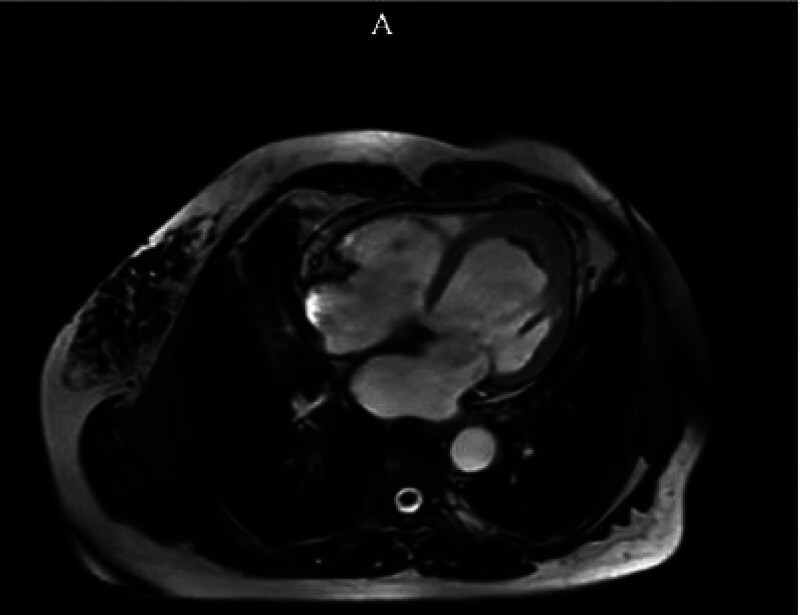
Local hypertrophy at the apex of the heart.

Cardiac MRI: local hypertrophy of the apex, no obvious abnormal signal, consistent with hypertrophic cardiomyopathy, enlarged left ventricle. It is suggested to combine with clinical practice (Fig. 4A).

Sinus CT: No abnormalities in bilateral paranasal sinuses were observed; bilateral inferior turbinates were slightly enlarged, and the nasal septum deviated.

The cardiac biopsy, eosinophilia-related genes and skin biopsy, and electromyography were refused by the patient.

### 2.3. Treatment protocols

With rash on limbs, the patient was diagnosed with solar dermatitis after a dermatological consultation and treated with oral ebastine, ketotifen, and topical mometasone furoate cream for external treatment.

After an ophthalmological consultation, the eyes were considered chronic uveitis and dry eye syndrome, being treated with tropicamide eye drops, tobramycin dexamethasone eye fluid, and polyethylene glycol eye drops. Meanwhile, salmeterol was inhaled to relieve asthma, third-generation cephalosporin was given for anti-infection treatment, and methylprednisolone 50 mg, cyclophosphamide 0.4 g for immunosuppression treatment. Patients agree with the above treatment plan. The symptoms got better after 2 days. The rash was significantly darker than before, wheezing was alleviated, and eosinophils returned to the average level.

After discharge, the patient continued to take methylprednisolone (50 mg) orally, and returned to the hospital for cyclophosphamide 0.4 g half a month later, having the clinical symptoms improved significantly – the rash subsided. Obviously, asthma did not recur and no notable abnormality was found in the whole EMG.

## 3. Discussion

EGPA, which is a rare autoimmune vasculitis involving small and medium blood vessels, still belongs to ANCA-associated vasculitis^[5]^ despite 60% of cases being no positive ANCA. With 3 natural courses, prodromal stage, eosinophil infiltration stage, and vasculitis stage,^[6]^ not all patients with EGPA show the characteristic progression of the disease, and it is most commonly associated with asthma, sinusitis, and peripheral eosinophilia in the peripheral blood. Although the lungs are the most commonly destroyed organ in this disease, it also may result in varying degrees of kidney, gastrointestinal, neurological, skin, or heart disease^.[7]^ Previous EGPA case reports show that cases confirmed by the involvement of these organs have existed, among which cardiopulmonary involvement was more common. However, no single case of long-term hemodialysis complicated by a rash of extremities was diagnosed.

Compared with granulomatosis with polyangiitis (GPA) and microscopic polyangiitis (MPA) also belong to ANCA-associated vasculitis, there exists a significantly lower frequency and severity of renal involvement in EGPA,^[8]^ uncommon renal insufficiency with only 5% of patients experiencing rapid progressive glomerulonephritis. Renal manifestations have been identified as independent predictors of EGPA mortality.^[9]^

During the review of the course of the disease, having a high incidence of rash, uremia dialysis patients did not attract enough attention at first, being considered that the rash and pruritus of limbs might be caused by secondary hyperparathyroidism, calcium, and phosphorus metabolism disorder. Calcification defense might be possible, but vascular calcification is not detected. Meanwhile, a history of asthma with frequent exacerbations and increased eosinophils was also consistent with the disease, and multiple previous cardiac echocardiography studies showed ventricular enlargement. However, cardiac echocardiography this time showed that the left ventricular apical area was thickened with decreased echo, thus considering the possibility of eosinophilic endocarditis? As the sporadic disease required a biopsy to confirm, the bone marrow biopsy, Epstein–Barr virus test, parasites test and others were performed to rule out diseases that may cause elevated eosinophils. A bone marrow smear examination suggested elevated eosinophils. However, the patient refused to complete eosinophilia-related genetic tests. Then, ANCA results showed positive P-ANCA and MPO-Ab. Hence, considering EGPA, studies showed that the best biopsy sites for observing vascular changes and granuloma formation are lungs, peripheral nerves, skin, gastrointestinal tract, and heart^,[4]^ since the EMG, as well as biopsy, were refused by the patient, the basis for the diagnosis of EGPA was insufficient. We improved cardiac MRI suggesting local apex hypertrophy, consistent with the manifestation of hypertrophic cardiomyopathy and left ventricular enlargement, as cardiac MRI – an significant noninvasive examination approach is suitable for patients suspected to be diagnosed with eosinophilic myocarditis in accordance with previous EGPA-related studies.^[10]^ Dennert et al reported that 38% of patients can still detect the pathologic change of the heart by echocardiography or cardiac magnetic resonance imaging (MRI) in the absence of symptoms and major ECG abnormalities,^[11]^ same with our case. It is a must for imaging tests to have MRI in that it is able to assess detailed anatomical descriptions of cardiac lesions.^[10]^ Eosinophilic infiltration and myocardial hypertrophy are the main manifestations of eosinophilic myocarditis. Although no myocardial biopsy was performed to determine whether this patient was infiltrated by eosinophils, MRI was also able to identify pathological changes in the myocardium.^[12]^

As the challengeable EGPA diagnosis may present variable clinical manifestations and not all patients with suspected EGPA have histological evidence, most EGPA cases are diagnosed and classified based on clinical manifestations and noninvasive examination results. Having a previous history of bronchial asthma with repeated lung lesions, the patient was admitted to our hospital for treatment, considering lung infection, and then discharged after the situation improved. Some other symptoms also existed – rash on limbs, increased eosinophils, hyperemia in both eyes considered chronic uveitis and dry eye syndrome, and positive P-ANCA and MPO-ANCA. Asthma with increased eosinophils is a major biomarker in the diagnosis of EGPA. EGPA consensus working group released the recommendations for patient assessment and management of EGPA in 2015, showing that positive MPO-ANCA is highly suggested for EGPA in the clinical context of asthma and eosinophils. Although no biopsy was performed, evidence of nervous system involvement was lacking, according to the classification criteria for vasculitis jointly developed by ACR/EULAR in 2022, the patient’s score is >6 points, and EGPA is considered for diagnosis. In addition, as multiple organ damages were involved, hormone combined with cyclophosphamide was applied for treatment. Studies have shown corticosteroids’ rapid and significant effect on EGPA-induced asthma and systemic symptoms^.[13]^ After the patient was treated with hormone + cyclophosphamide, the rash of extremities was quickly relieved, and eosinophils also returned to normal levels. During the follow-up visit to our hospital 2 weeks later, the patient’s rash had obviously subsided, asthma did not recur, and the outpatient electromyogram showed no obvious peripheral neuropathy, suggesting a notably effective treatment. However, long-term follow-up is a must.

It is obvious in studies that hemodialysis itself does not seem to be prone to interfere with disease activities as well as arouse their outbreak.^[14]^ When the patient was diagnosed with chronic renal failure in the local hospital before, the eosinophils were not high without developing asthma as well, and it was unclear whether there were abnormal ANCA results. Symptoms, such as rash and asthma, did not appear until the dialysis was conducted for 2 years, conforming to the findings that hemodialysis did not make the exposure to immune deficiencies faster. In the case described, the onset of the patient’s asthma and rash may have originated from eosinophilia. Terrier et al have demonstrated that eosinophilia is related to active activities,^[15]^ and the appearance of the eosinophilia and rash seems to be supportive of this point of view.

This case highlighted the difficulties clinicians faced up in making the diagnosis and the diagnostic approaches available for suspected EGPA were reviewed. A great understanding of this disease could facilitate early diagnosis to reduce mortality and preserve organ function. Studies have shown that all eosinophilic myocarditis requires timely treatment so as to prevent myocardial scar formation and fibrotic changes^,[7]^ which brings about a therapeutic dilemma for doctors in that treatment with glucocorticoids can delay confirmation of an underlying diagnosis in the long run. At the same time, it shall improve patients’ clinical symptoms in the short term. It is tricky for EGPA to diagnose due to its multiple organ involvement and manifestations. There are no routinely used biomarkers that can distinguish EGPA from other diseases until now.

## 4. Conclusion

A family of rare ANCA-associated vasculitis, EGPA refers to a combination of eosinophilic hyperplasia and vasculitis causing damage to the body simultaneously, among which eosinophilic granuloma formation is usually dependent on biopsy. In this case, the patient has clinical features of asthma and increased eosinophilia combined with positive P-ANCA and MPO-ANCA, being highly suspected of EGPA. However, any 4 of the diagnostic criteria did not match with that proposed by the American College of Rheumatology in 1990 (including asthma-like symptoms, eosinophilia, nonfixed pulmonary infiltrates, single or multiple neuropathies, paranasal sinus lesions, and biopsy showing extravascular eosinophilic infiltration).^[16]^ Thus, the failure to diagnose suggests a difficult early diagnosis and treatment of EGPA. Most patients failed to reach the diagnostic criteria because of no involvement of multiple organs in the early stage, while the patient having 5 years of dialysis, which seemed to have no risk of interfering with disease activities and outbreak without a diagnosis of EGPA during the period, was confirmed finally on account of the rash of limbs and abnormal color Doppler ultrasound caused by high eosinophils. This has aroused our profound thought that it is a lack of understanding of EGPA for our clinicians and that the ANCA test shall be put on the high agenda for patients presenting with kidney failure at first. Meanwhile, the neglected immune monitoring for patients with dialysis tells us that it is of great significance for this kind of patient to have immune monitoring in the early diagnosis of EGPA, so that the misdiagnosis and missed diagnosis shall be reduced and early treatment shall be conducted to improve their prognosis^.[17]^

The report of this study is in line with the Care Guide.

## Acknowledgments

Thanks to Yibin Second People’s Hospital for their support to write this case report.

## Author contributions

**Writing – original draft:** Lingshan Zhao.

**Writing – review & editing:** Chenli Zhang.
